# Lactic acid bacteria: promising supplements for enhancing the biological activities of kombucha

**DOI:** 10.1186/s40064-015-0872-3

**Published:** 2015-02-24

**Authors:** Nguyen Khoi Nguyen, Ngan Thi Ngoc Dong, Huong Thuy Nguyen, Phu Hong Le

**Affiliations:** Center of Research and Technology Transfer, International University, Vietnam National University, Ho Chi Minh City, 70000 Vietnam; School of Biotechnology, International University, Vietnam National University, Ho Chi Minh City, 70000 Vietnam; Department of Biotechnology, Faculty of Chemical Engineering, University of Technology, Vietnam National University, Ho Chi Minh City, 70000 Vietnam

**Keywords:** Antibacterial activity, 2,2-diphenyl-1-picrylhydrazyl, Fermented tea, Glucuronic acid, Kombucha

## Abstract

Kombucha is sweetened black tea that is fermented by a symbiosis of bacteria and yeast embedded within a cellulose membrane. It is considered a health drink in many countries because it is a rich source of vitamins and may have other health benefits. It has previously been reported that adding lactic acid bacteria (*Lactobacillus*) strains to kombucha can enhance its biological functions, but in that study only lactic acid bacteria isolated from kefir grains were tested. There are many other natural sources of lactic acid bacteria. In this study, we examined the effects of lactic acid bacteria from various fermented Vietnamese food sources (pickled cabbage, kefir and kombucha) on kombucha’s three main biological functions: glucuronic acid production, antibacterial activity and antioxidant ability. Glucuronic acid production was determined by high-performance liquid chromatography–mass spectrometry, antibacterial activity was assessed by the agar-well diffusion method and antioxidant ability was evaluated by determining the 2,2-diphenyl-1-picrylhydrazyl radical scavenging capacity. Four strains of food-borne pathogenic bacteria were used in our antibacterial experiments: *Listeria monocytogenes* ATCC 19111, *Escherichia coli* ATCC 8739, *Salmonella typhimurium* ATCC 14028 and *Bacillus cereus* ATCC 11778. Our findings showed that lactic acid bacteria strains isolated from kefir are superior to those from other sources for improving glucuronic acid production and enhancing the antibacterial and antioxidant activities of kombucha. This study illustrates the potential of *Lactobacillus casei* and *Lactobacillus plantarum* isolated from kefir as biosupplements for enhancing the bioactivities of kombucha.

## Background

Lactic acid bacteria (LAB) are important microbes that have long been used in both traditional and modern industrial food fermentation. As well as adding flavor, many food products fermented by LAB (e.g. yogurt, cheese, pickled cabbage and kefir milk) are believed to convey health benefits (Dufresne and Farnworth 2000). These proposed health benefits include stimulation of the human immune system and antimicrobial activity. Adding LAB to food products can also improve biological activities such as food preservation, wheat bread and cocoa fermentation, and D-saccharic acid 1,4 lactone (DSL) production in kombucha (KBC) (Masood et al. 2011; Rollán et al. 2010; Yang et al. 2010; Kresnowati et al. 2013). The addition of LAB to food products has therefore increased in recent years.

KBC is a sweetened black tea fermented by a symbiotic colony of bacteria and yeast. The interaction of these microorganisms results in a floating cellulose layer on the surface of the fermented tea. The longer the fermentation, the thicker the layer becomes. The bacterial component of KBC cultures has not been extensively studied but is known to comprise several species, including acetic acid bacteria (AAB). Recently LAB were reported to comprise up to 30% of the bacterial population of KBC cultures (Marsh et al. 2014; Yang et al. 2010). One report investigated the interaction between LAB from kefir milk (a fermented milk drink made using kefir ‘grains’ as a yeast/bacterial fermentation starter) and AAB from KBC with respect to growth rate, biomass and secondary metabolites. LAB were shown to improve the survival of AAB, and this combination was found to be the optimal mixed culture to enhance DSL production in KBC (Yang et al. 2010). Furthermore, the vitamin B complex secreted by AAB (in particular *Acetobacter* sp*.*) provides a favorable environment for the growth of other LAB and yeast in kefir grains (Leite et al. 2013).

Although much work has been done to evaluate the positive stimulation in growth rate, biomass and secondary metabolites provided by the co-cultures of *Lactobacillus* spp*.* in KBC, the human health benefits have not been fully explored. Therefore, we focused on the significance of *Lactobacillus* spp. in mixed culture KBC in enhancing its three important biological functions: glucuronic acid (GlcUA) production, antibacterial activity and antioxidant ability. In this study, we isolated *Lactobacillus* strains from kefir, pickled cabbage and KBC, and cultured these with the KBC layer in the sweetened black tea medium. We assessed glucuronic acid production, antibacterial activity and antioxidant ability. We believe that the results of this study will enable the beverage industry to produce higher quality healthy fermented tea.

## Results and discussion

### Isolation and identification of *Lactobacillus* spp*.* from kefir grains

The non-spore forming Gram-positive isolated bacterial strains showed rod shaped morphology under a microscope, and formed round milky colonies on Man, Rogosa and Sharpe (MRS) agar. Two strains of *Lactobacillus* spp*.* were isolated from pickled cabbage (*lac1* and *lac2*), one from KBC (*lac3*), and two from kefir milk (*lac4* and *lac5*).

### Evaluation of glucuronic acid production

**Figure 1:**
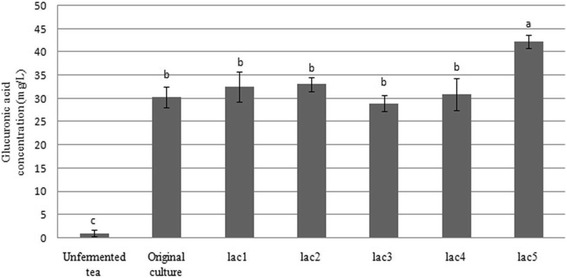
**Glucuronic acid production of kombucha fermented by the kombucha layer and supplementation with** ***Lactobacillus*** **spp. (** ***lac1*** **–** ***lac5*** **).** Original kombucha: fermented sweetened black tea only (kombucha layer). Unfermented tea: sweetened black tea. The significant differences between samples are indicated by superscript letters (a–c) with p ≤ 0.05.

Interest in GlcUA production in food products has increased significantly over the last decade. Many studies have evaluated different methods of enhancing GlcUA production by optimizing fermentation conditions or improving the culture medium. However, little is known about the effect of supplemented *Lactobacillus* spp. on GlcUA production in KBC cultures. In this study, high-performance liquid chromatography-mass spectrometry (HPLC-MS) was employed to quantify the GlcUA concentration in KBC layer cultures supplemented with each *Lactobacillus* sp. (*lac1*–*lac5*). The results are presented in Figure 1. The level of this organic acid was moderate in the presence of strains *lac1*–*lac4*, but sharply increased in the presence of strain *lac5*.

On the fifth day of fermentation, the combination of strain *lac5* and the KBC layer produced 39.6% more GlcUA than the original culture (42.3^a^ mg/L compared with 30.3^b^ mg/L). Thus, strain *lac5* was more effective at stimulating GlcUA production than the other LAB strains studied.

The combination of *Lactobacillus* spp. with AAB in KBC enhanced the DSL concentration, which determines the GlcUA level in the glucuronate pathway (Yang et al. 2010). This could be a possible explanation for the improvement in GlcUA production conferred by *Lactobacillus* spp. in our study. The GlcUA values obtained in our study were higher than those reported by other studies (i.e. 10 mg/L and 3.39 mg/L) despite using similar fermentation conditions (Blanc 1996; Loncar et al. 2000). Experimental results may vary because of the duration of fermentation or variations in the symbiotic cultures used, which may in turn affect the GlcUA concentration (Mayser et al. 1995). Further research is needed to ascertain whether strain *lac5*can be applied to KBC to improve any health benefits.

### Evaluation of antibacterial activity

The antibacterial activity of KBC was also investigated using the filtrate from 5-day-fermented tea broth as the testing sample. The agar well diffusion method was employed. Four pathogenic microbes, *Listeria monocytogenes* ATCC 19111, *Escherichia coli* ATCC 8739, *Salmonella typhimurium* ATCC 14028 and *Bacillus cereus* ATCC 11778, were grown to confluence on agar plates, after addition of the testing sample to the well, the diameter of inhibition (the halo zone) was measured for each bacteria strain. Table 1 shows clear inhibition of all four pathogenic microbes by the KBC filtrate. Mixed cultures of the KBC layer with different *Lactobacillus* spp*.* (*lac1*–*lac5*) were also tested; *lac3* gave the largest halo zone for all four bacteria, with measurements of 8.50, 6.75, 9.15 and 9.00 mm for *E. coli, B. cereus, S. typhimurium* and *L. monocytogenes*, respectively. In contrast, the original KBC culture showed the lowest effect on the test sample. The halo zone remained unchanged, thus the diameter was recorded as 0 mm. The conventional culture of KBC also showed antibacterial activity against many types of unexpected bacteria (data not shown). This antibacterial activity is mediated by the acetic acid and ethanol excreted by the yeast and AAB or from cellulose membrane-producing bacteria.

**Table 1:** **Antibacterial assay of kombucha fermented by the kombucha layer supplemented with** ***Lactobacillus*** **spp. (** ***lac1*** **–** ***lac5*** **)**

**Diameter of the Halo zone (mm)**				
	*E.c*	*B.c*	*S.t*	*L.m*
U.tea	0	0	0	0
O.cul	5.50 ± 0.7	1.75 ± 1.0	7.05 ± 0.6	3.75 ± 0.3
*Lac1*	6.75 ± 1.0	3.50 ± 0.7	7.50 ± 0.7	7.00 ± 0.7
*Lac2*	4.50 ± 0.7	4.50 ± 1.4	3.00 ± 2.8	5.75 ± 0.7
*Lac3*	8.50 ± 0.7	6.75 ± 1.0	9.15 ± 0.5	9.00 ± 0.7
*Lac4*	2.00 ± 1.4	6.25 ± 0.3	5.75 ± 0.3	7.50 ± 0.7
*Lac5*	6.50 ± 0.7	3.25 ± 0.3	6.75 ± 0.3	6.00 ± 1.4

U.tea: unfermented sweetened black tea; O.cul: original culture from the fermentation of sweetened black tea only (kombucha layer). *L.m*: *Listeria monocytogenes* ATCC 19111, *E.c*: *Escherichia coli* ATCC 8739, *S.t*: *Salmonella typhimurium* ATCC 14028, *B.c*: *Bacillus cereus* ATCC 11778.

The finding that supplemented *lac3* showed an improvement in kombucha’s natural antibiotic effects highlights other advantages in supplying these bacteria to fermented tea in addition to enhancing its biological activities. To further our understanding, specific experiments need to be conducted to evaluate the antibacterial activities of this *Lactobacillus* strain individually. This should help to identify the main antibiotic reagents in the metabolites of this organism. These findings are promising in the search for biosupplements that can improve the gastrointestinal system and potentially confer other probiotic properties.

### Evaluation of antioxidant activity

The antioxidant activity of KBC was then investigated. Table 2 shows the antioxidant potential of KBC, the original culture and the mixed culture with different *Lactobacillus* spp*.* against the oxidant 2,2-diphenyl-1-picrylhydrazyl (DPPH). The antioxidant activity on DPPH radical scavenging may be dependent on the hydrogen-donating ability of a particular culture. The DPPH scavenger capacity of the tea samples was compared with that of vitamin C, a known antioxidant. The highest level of DPPH discoloration was observed with vitamin C (93.00%) and the lowest level was observed with the unfermented tea sample (62.75%). The mixed cultures of the KBC layer with different *Lactobacillus* spp. showed antioxidant abilities ranging from 71.00% to 89.50%.

**Table 2:** **Antioxidant assay of kombucha fermented by the kombucha layer and supplementation with strains of** ***Lactobacillus*** **spp. (** ***lac1*** **–** ***lac5*** **)**

**Antioxidant values (%) (percentage of DPPH inhibition)**	
Vitamin C	93.00 ± 1.4^a^ (%)
*lac1*	71.00 ± 1.4^c^ (%)
*lac2*	71.75 ± 2.4^c^ (%)
*lac3*	81.25 ± 1.7^b^ (%)
*lac4*	89.50 ± 0.7^a^ (%)
*lac5*	78.00 ± 2.1^bc^ (%)
Unfermented tea	62.75 ± 3.1^d^ (%)
Original culture	79.75 ± 0.3^b^ (%)

Original Culture: traditional sweetened black tea fermented (kombucha layer only). Unfermented tea: sweetened black tea. The significant differences between samples are indicated by superscript letters (a–d) with p ≤ 0.05.

**Figure 2:**
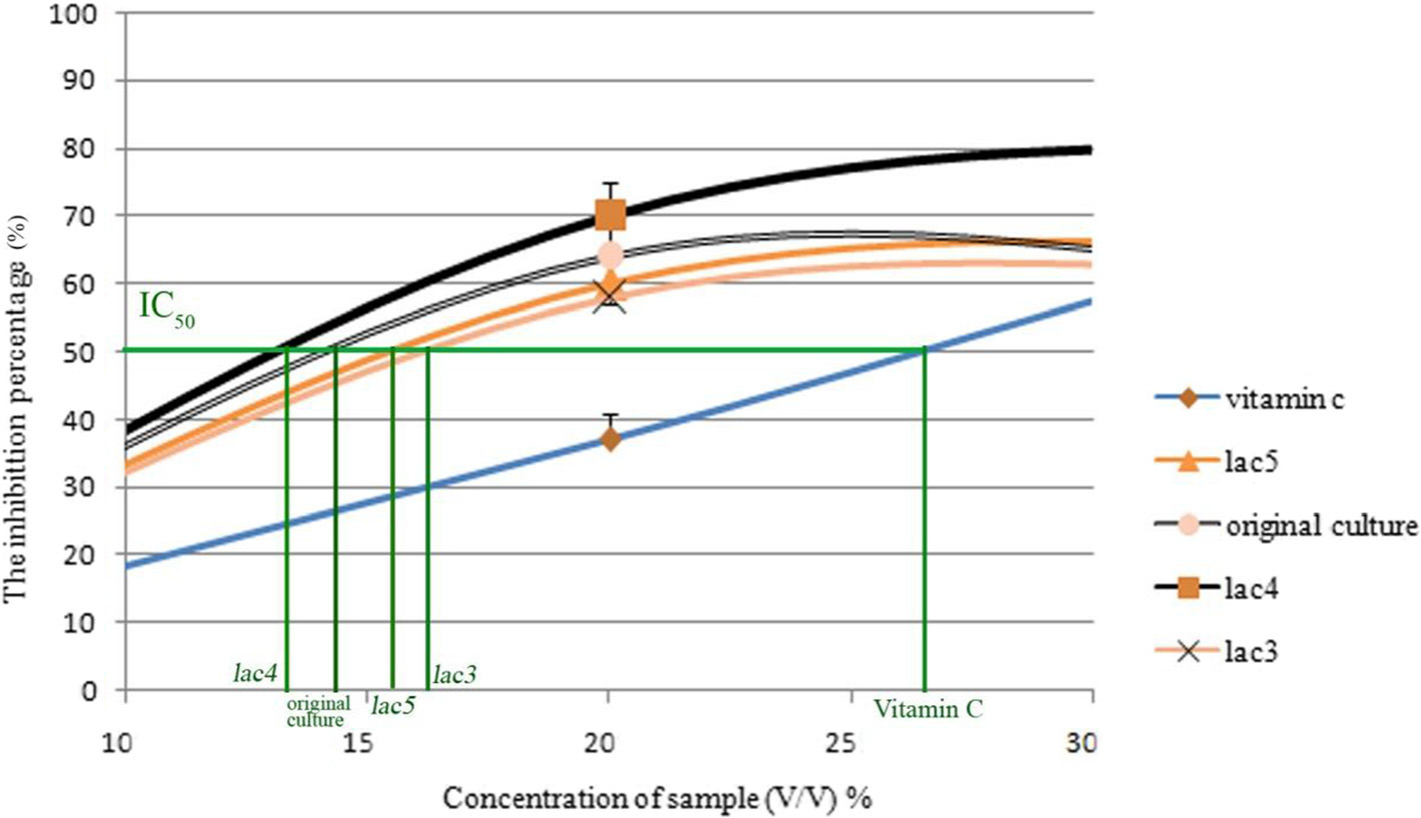
**Plot of the DPPH free radical-scavenging activity of each fermented tea sample at various concentrations.** The amount of sample that inhibits 50% of the DPPH indicates its IC_50_ value, which is inversely proportional to its antioxidant effect.

In contrast, the IC_50_ values of *lac3*, *lac4*, *lac5* and the original sample were lower than the IC_50_ value of vitamin C, which means less of each sample was needed to reduce half of the DPPH solution compared with the positive control (Figure 2). The IC_50_ value of vitamin C was more than 20% of the sample concentration; while the IC_50_values of *lac*3, *lac4*, *lac5* and the original culture were lower than 20%. The antioxidant properties of KBC may be high because vitamin C, vitamin B and DSL are synthesized during fermentation. However, our results are lower than those reported in lemon-balm KBC, in which the antioxidant activity was higher than 90% (Velićanski et al. 2007). This difference may reflect differences in the amounts used, and the different antioxidant compounds present in black and lemon-balm tea.

### Molecular identification of *Lactobacillus* strains

The results of DNA sequence analysis revealed that *lac3* and *lac4* are *Lactobacillus plantarum* and *lac5* is *Lactobacillus casei*. These strains have been shown to improve certain biological activities of KBC, such as GlcUA production, antibacterial activity and antioxidant activity. In addition, *L. casei* has been reported to strengthen the immune system and prevent *Candida albicans* infection in humans*. L. plantarum* has been considered a therapeutic bacterium because of its DNA cellular protection and anticancer ability (Masood et al. 2011). Our results emphasize the advantages of *L. plantarum* and *L. casei* isolated from kefir compared with other *Lactobacillus* sp. from other sources, confirming our previous findings that these LAB strains improve the biological function of KBC (Nguyen et al. 2014).

## Conclusions

In this study, *Lactobacillus* spp. isolated from KBC and kefir, in particular some strains of *L. casei* and *L. plantarum*, show improvements in the GlcUA concentration and the antibacterial and antioxidant activities when used to supplement KBC compared with the original KBC. This enhancement of the biological functions of KBC may increase the popularity of these fermented drink products. These findings once again demonstrate the value of *Lactobacillus* strains in the food and beverage industry. However, further investigations into the mechanisms involved in biological metabolite production are necessary. These preliminary findings are a significant step towards producing fermented tea with improved health benefits.

## Methods

### Chemicals, raw materials and microbial culture preparation

Pickled cabbage, KBC layers and kefir grains were obtained from a local market in Ho Chi Minh City (Vietnam). *Listeria monocytogenes* ATCC 19111*, Escherichia coli* ATCC 8739, *Salmonella typhimurium* ATCC 14028 and *Bacillus cereus* ATCC 11778 were provided by Microbiologics Company (St. Cloud, MN, USA).

Lactobacilli, MRS medium and tryptone soybean agar were provided by Himedia Company (Mumbai, India). The tea substrate used in this study was Lipton black tea, a product of Unilever Company. Glucuronic acid (G5269-10G, Sigma–Aldrich, St. Louis, MO, USA), was used as a standard for HPLC-MS.

DPPH (Sigma–Aldrich) was used in the antioxidant assay. An HPLC system (Agilent 1200) equipped with a mass spectrometer (micrOTOF-Qll, Bruker) and ACE3 C-18 column (4.6 × 150 mm) was used to determine the GlcUA concentration.

### Preparation of sweetened black tea and KBC

One liter of autoclaved sweetened black tea containing 100 g sucrose and 1 g Lipton black tea extract in boiling water was prepared. Fermented tea was cultured in the sweetened black tea medium by adding 5 g of the wet KBC layer to 100 mL of the total volume.

### Preparation of original KBC culture and the mixed cultures

The biomass of the various *Lactobacillus* spp. was removed from the enrichment medium by centrifugation at 4000 × *g* for 15 min at 4°C before culturing in the sweetened black tea. The mixed cultures contained the KBC layer and *Lactobacillus* spp., and the original culture contained only the KBC layer. Culturing was performed at pH 5 for 5 days at 30°C. The space remaining above the culture was 60% of the total volume of the container. Unfermented sweetened black tea was used as a negative control.

### Isolation of *Lactobacillus* spp

Kefir grain was maintained by serial subculture in defatted milk at 25°C for 3 days. The bacterial strains were selected from the homogenous milk based on the growth conditions and their morphology on MRS agar medium. Broth samples from KBC and pickled cabbage were ten-fold serially diluted and then streaked onto MRS agar plates. Single colonies were observed on MRS after incubation at 37°C for 72 h under anaerobic conditions. Pure cultures were maintained, with the MRS medium being refreshed weekly, for further experiments.

### Quantification of glucuronic acid by HPLC-MS

Before the fermented tea samples were injected into HPLC vials, they were loaded onto an SPE C18 column and passed through a millipore filter (0.45 μm). Then, 20 μL of the filtrate was pumped to an HPLC system equipped with a mass spectrometer and ACE3 C-18 column for analysis. Formic acid (0.1%) in deionized water was used as the mobile phase and formic acid (0.1%) in methanol was used as the stationary phase. The flow rate was adjusted to 0.5 mL/min at room temperature (23–25°C) for the 210-nm wavelength. The resolution peaks recorded on the HPLC chromatogram report were relative to the retention time of the GlcUA standard. The concentrations were quantified from standard curves and multiplied by the dilution factors. The experiment was completed at the Central Laboratory for Analysis at the University of Science, Vietnam National University, Ho Chi Minh City, Vietnam.

### Antibacterial activity assay

A clear 5-day-fermented tea broth was obtained by passing the liquid cultured sample through a 0.22-μm pore size filter paper. The filtrate was then used as the testing sample. *Listeria monocytogenes* ATCC 19111, *Escherichia coli* ATCC 8739, *Salmonella typhimurium* ATCC 14028 and *Bacillus cereus* ATCC 11778 were grown to confluence on solid agar and the agar diffusion method was performed as described by Irshad et al. (2012). Briefly, the test sample was added to the center of the agar plates with confluent bacterial growth and the diameter of inhibition (the halo zone) was measured for each bacterium for each of the test samples. The test samples included the unfermented tea; the original culture of KBC fermented by the KBC layer, and mixed cultures of the KBC layer with different *Lactobacillus* spp*.* (*lac1*–*lac5*). The diameters of the halo zones were recorded after 24 h incubation at 37°C.

### Antioxidant activity and IC_50_ measurement

Screening of the DPPH free radical-scavenging activities of the fermented tea samples was carried out according to Küçükboyaci et al. (2012) with a slight modification. Briefly, 0.5 mL fermented tea broth was mixed with 1.5 mL ethanolic DPPH solution (250 μM). A mixture of absolute ethanol (1.5 mL) and unfermented tea broth (0.5 mL) served as a negative control. A 100-μM vitamin C solution was used as a positive control. The absorbance was measured at a wavelength of 517 nm, and the percentage inhibition was calculated using the following equation.

$$ x\%=100-\left[\frac{\left({\mathrm{Abs}}_{\mathrm{sample}}-{\mathrm{Abs}}_{\mathrm{blank}}\right) \times 100}{{\mathrm{Abs}}_{\mathrm{control}}}\right] $$

For IC_50_ determination, a sample of the fermented tea broth solution was diluted to a range of concentrations (20%, 40%, 60%, 80% and 100%). Each concentration was reacted with DPPH following the above procedure, resulting in a five-point graph for each sample. The IC_50_ is defined as the concentration of sample required to scavenge 50% of the DPPH. The experiment was performed in triplicate for each sample, and the results were calculated as a percentage decrease from the control values and compared by one-way ANOVA. A difference was considered statistically significant if p ≤ 0.05.

### Molecular identification of *Lactobacillus* spp*.* strains

The selected *Lactobacillus* strains, which confer positive stimulation of KBC biological activities, were sequenced by NK-Biotek Company (Ho Chi Minh City, Vietnam), and the obtained sequences were analyzed and compared using BLAST software (http://blast.ncbi.nlm.nih.gov/Blast.cgi).

## Abbreviations

AAB: Acetic acid bacteria
DSL: D-saccharic acid 1,4 lactone
DPPH: 2, 2-diphenyl-1-picrylhydrazyl
MRS: de Man, Rogosa and Sharpe
GlcUA: Glucuronic acid
KBC: Kombucha
LAB: Lactic acid bacteria
